# A First Experience†We publish this case of Dr. Gustin’s, as it teaches a lesson by its failures and its final success. After trying empirical measures, Dr. G. had the good sense to return to homœopathic prescriptions and was well rewarded. His failure to do any good with Morphine should teach a lesson. It is just in this way that eclectic practice grows up; one fails after repeated hasty and ill-considered prescriptions. Then he says “something must be done,” and goes off into eclectic practice. Prescribe slowly, carefully, and do not change your remedy, save for one *better* indicated! Give each prescription *time* to show its work.—Eds.

**Published:** 1888-04

**Authors:** F. M. Gustin

**Affiliations:** Union City, Ind.


### A FIRST EXPERIENCE.†

Editors Homœopathic Physician:—I have been treating of late some rather interesting cases with Sulphur$^{cm}$. which I will arrange later for publication, when they shall have been cured a sufficient length of time to justify a report. I concluded meanwhile to write you an account of the first case in which I witnessed the marvelous effects of the curative remedy in a dangerous disease.

It was early in my professional work that, on the 17th of August, an elderly gentleman came into my office and requested me to see his youngest daughter, then about fourteen years of age.

She had had, the week before, an attack of dysentery, which an eclectic physician had suppressed with walnut bark, and had apparently cured her; but the pains and tenesmus had returned more severely than ever. There was absolutely no stool, only

† We publish this case of Dr. Gustin's, as it teaches a lesson by its failures and its final success. After trying empirical measures, Dr. G. had the good sense to return to homœopathic prescriptions and was well rewarded. His failure to do any good with Morphine should teach a lesson. It is just in this way that eclectic practice grows up; one fails after repeated hasty and ill-considered prescriptions. Then he says "something must be done," and goes off into eclectic practice. Prescribe slowly, carefully, and do not change your remedy, save for one *better* indicated! Give each prescription *time* to show its work.—Eds.

a *very scanty*, *white*, *glairy* substance, which was allowed to pass on a cloth as she lay in bed. She would lie upon her back, draw her knees up to her chin, and strain as though she thought she must have a stool, and then say, " Why won't it pass away from me?"

Her tongue was coated a yellowish brown, mostly in centre, with border and tip red; thirst for rather large drinks of water, and crampy pains in the limbs; was rather cross and fretful or changeable in humor.

I prescribed for her for five days before I succeeded in finding the remedy, although I was hunting for it day and night. I used *Bell.*, *Coloc.*, *Nux v.*, *Cham.*, *Ars.*, and perhaps other remedies; about the third day I thought I had the case under control with Chamomilla, but she was soon as bad as ever, in fact, was getting worse all the time.

She would frequently exclaim, "Oh! I know I will die if I don't soon get better." Would often say, " Oh! my bowels will burst." This symptom is given in some repertories as characteristic of Arsenicum, which was given in different potencies without effect, as might have been expected if the thirst had been kept in mind. She would often say her bowels felt as though they were all full of sore pimples.

On the night of the third day I gave her a large dose of olive oil with hope that it might possibly soothe the bowels and give her some rest; and she did become quiet, and had a good sleep for two hours, after which she was the same as before.

On the fourth day retention of urine came, giving her much additional pain. This was relieved by placing a flannel, wrung out of hot water, over the bladder. Patient kept on getting no better, and on the fourth night I gave her small doses of first trituration of Morphine, which seemed to keep her quiet during the night.

On entering the room next morning her oldest sister said my powders had acted very nicely, but a glance at patient's tongue showed me they were doing bad work; the coating was thicker, tongue becoming very dry, thirst increasing, and every indication of an unfavorable termination in a short time. Soon the urine was suppressed again, and this time could not be relieved as before.

A peculiar mental symptom* was that "some one of the family

---

* Mental symptoms are our best guide; perhaps had this one been studied, the proper remedy would have been found. Patients think they have lost the affection of friends, is Aur. and Hura; think they are despised, is Arg-n. and Lac-can.—Eds.

was mad at her;" it troubled her very much during the fourth day, then disappeared. If told by every one that none were mad at her it would only satisfy her for a short time, but this symptom disappeared before the curative remedy was given.

For two days I had been studying *Sulphur* for the case, but could not see it clearly indicated.

There was nothing in appearance of the patient to suggest the remedy, but from the appearance of the tongue, the sensation as of pimples in the bowels with tenderness to touch, the tenesmus of the rectum and bladder, and, perhaps, other points which I cannot remember now, and remembering she had told me the trouble first began in the morning, I concluded that the only thing that could offer any hope was to give one dose of Sulphur and wait six hours.

The medicine was a graft of the CM potency which I had received from Dr. Z. Hockett, of Anderson, Indiana, that veteran homœopath of our State. I received it by letter, in a pasteboard vial which was mashed in the letter. I put the pellets in a vial, dissolved them with water condensed on a piece of glass over a teakettle, and filled the vial with alcohol. I gave the patient a single powder of this about nine o'clock, and left to return at four.

Within half an hour after taking the medicine she passed water easily, had a natural fecal stool, and slept well. At four o'clock I found her with moist tongue, free from pain, with some tenderness of bowels, but in good spirits. She kept her bed for a few days but recovered without any more medicine, and when her father called, two months later, he said she had not looked so well for three years.

This was my first experience with a "fluxion potency," and ever since then I feel I have a "friend in Sulphur$^{cm}$."

F. M. Gustin, M. D.

Union City, Ind.

